# Supplementary material for: A recurring disease outbreak following litchi fruit consumption among children in Muzaffarpur, Bihar—A comprehensive investigation on factors of toxicity
Source: PLoS One. 2020 Dec 31;15(12):e0244798. doi: 10.1371/journal.pone.0244798 (PMC7774918; doi:10.1371/journal.pone.0244798)
Supplement: Attachment — Submitted filename: Review plos one.docx [file pone.0244798.s001.docx]

Sinha et al has reported the work titled “A recurring outbreak of litchi fruit consumption among children of Muzaffarpur, Bihar”. Authors have tried to identify toxic compounds in fruit litchi and in urine samples obtained from children. Authors found MCPG in litchi samples and identified DEP in urine samples. In addition to these, they performed acetylcholinesterase biochemical assays. The present report is important study as litchi outbreak occurs every year in Bihar due to its consumption.

1. MCPG is a well reported metabolite in litchi. I was just wondering if authors found any other toxic compounds in the litchi fruit.
2. Did authors find any OP pesticides in litchi fruit? If yes what are they?
3. I would suggest quantifying the MCPG in the litchi samples and quantify DEP in urine samples.
