# Supplementary material for: A recurring disease outbreak following litchi fruit consumption among children in Muzaffarpur, Bihar—A comprehensive investigation on factors of toxicity
Source: PLoS One. 2020 Dec 31;15(12):e0244798. doi: 10.1371/journal.pone.0244798 (PMC7774918; doi:10.1371/journal.pone.0244798)
Supplement: Attachment — Submitted filename: PONE-D-20-25356 - Responces.docx [file pone.0244798.s002.docx]

**Response to reviewer comments**

Editor Comments

**Editor Comment 1:**

Please ensure that your manuscript meets PLOS ONE's style requirements, including those for file naming.

**Reply**: Manuscript has been prepared as per the journal requirements

**Editor Comment** 2:

i) Please provide additional details regarding participant consent. In the ethics statement in the Methods and online submission information, please ensure that you have specified what type you obtained (for instance, written or verbal, and if verbal, how it was documented and witnessed). If your study included minors, state whether you obtained consent from parents or guardians. If the need for consent was waived by the ethics committee, please include this information.
ii) ii) Once you have amended this/these statement(s) in the Methods section of the manuscript, please add the same text to the “Ethics Statement” field of the submission form (via “Edit Submission”).

**Reply:**

i) Written informed consent was obtained for all the children from their parents and all experiments were conducted under the institutional ethical guidelines by Balaji Utthan Sansthan, Patna, Bihar, India.

We confirm that all the experimental protocols and procedures were approved by a Balaji Utthan Sansthan institutional committee, Balaji Utthan Sansthan, Patna, Bihar, India.

ii) The statement has been amended into the manuscript and also in the submission form as per the suggestions.

**Editor Comment** 3:

We note that Figure [1] in your submission contain [map/satellite] images which may be copyrighted. All PLOS content is published under the Creative Commons Attribution License (CC BY 4.0), which means that the manuscript, images, and Supporting Information files will be freely available online, and any third party is permitted to access, download, copy, distribute, and use these materials in any way, even commercially, with proper attribution. For these reasons, we cannot publish previously copyrighted maps or satellite images created using proprietary data, such as Google software (Google Maps, Street View, and Earth). For more information, see our copyright guidelines: <http://journals.plos.org/plosone/s/licenses-and-copyright>.

**Reply**: Figure 1 has been removed as suggested.

**Editor Comment** 4:

Please upload a new copy of Figures 2 and 3 as the detail is not clear.

**Reply**: Original and clear images for figures for 2 and 3 are uploaded.

**Reviewer Comments**

***Reviewer #1:***

**Reviewer Comment 1**:

The English language and grammar are very poor throughout the manuscript.

**Reply:** The whole manuscript was checked for English language and grammar errors and revised accordingly and also the revised manuscript was proofread by a native English speaker and corrected by Grammarly software finally.

**Reviewer Comment 2:**

There are a lot of sentences in the introduction which are not properly cited.

**Reply:** We thank the reviewer for the identification of errors and the same has been rectified in the revised manuscript.

**Reviewer Comment 3:**

The content of the introduction section should be improved focusing more on MCPG and pesticide toxicity.

**Reply:** Changes and additions with regards to MCPG and pesticide toxicity have been revised into the manuscript.

**Reviewer Comment 4:**

The author should increase the sample size of the urine and plasma samples for this study which can be statistically significant.

**Reply:** The samples were collected in the year 2014 during the outbreak as a part of the present investigative study, which has shown interesting findings and further research with large sample size is required.

**Reviewer Comment 5**:

Section 2.6, 2.8 and 2.9 can be merged.

**Reply**:

Section 2.6, 2.8 and 2.9 are merged as per the reviewer suggestions (Page NO: 8-9)

**Reviewer Comment 6:**

The urine and plasma samples were not subjected for protein precipitation prior extraction. Any explanation?

**Reply:** The method employed in our study was a reported method developed by the CDC Atlanta for the analysis of pesticide metabolites in urine samples.

**Reviewer Comment 7:**

The quality of all the images provided are very poor. please improve.

**Reply**: Image quality has been improved and new images with high resolution are included in the revised manuscript.

***Reviewer #2:***

**Reviewer Comment 1**

There are numerous grammatical and typo errors, and the explanations are not clear in some places. For example, in the first paragraph of the introduction “In India, a total of 68.49 metric tons were produced, and 25.40 metric tons were imported in 2012-2013” this sentence is for litchi production or pesticide consumption it is not clear.

**Reply:** The details mentioning about production and export was with regard to litchi fruit. The same is now clearly mentioned in the revised manuscript. Also, as per the reviewer's suggestions, manuscript was checked for any ambiguous sentences and revised.

**Reviewer Comment 2**

In the second paragraph of the introduction, the author has mentioned the first outbreak year as 2012 instead of 2011 which is not matching what has mentioned in the abstract.

**Reply:** The typographic error of 2012 was corrected to 2011 which is the first outbreak occurred in Bihar. (Page -3, Paragraph 2)

**Reviewer Comment 3**

In the third paragraph of the introduction, the first line of the paragraph “plant extracte” should be replaced with “plant extract”.

**Reply:** Typographical error of plant extracte to plant extract has been corrected as per the reviewer suggestion. (Page -3, Paragraph 3)

**Reviewer Comment 4**

In the fourth paragraph of the introduction the full name of KDKM medical clinic and SKMCH hospital should be mentioned.

**Reply:** The full name of KDKM **(**Krishnadevi Deviprasad Kejriwal Maternity Hospital) and SKMCH (Shri krishna Medical College & Hospital) are provided in the revised manuscript. (Page 4, Paragraph 1)

**Reviewer Comment 5**

In the fifth paragraph, the seventh line “The sex proportion was seen as 1.2:1 male to female”. It is not clear for which place this data has been mentioned. In the 8th line, reference 14 is not in a proper format.

**Reply:** The necessary modified indicating the proportions and referencing in proper format were made in the revised manuscript as per the reviewer suggestions. (Page 4, Paragraph 1)

**Reviewer Comment 6**

In the material method section, Acetyl-cholinesterase estimation, the author should mention the full name of SLK diagnostics

**Reply:** The name SLK diagnostics mentioned in the manuscript represents the name and the diagnostic laboratory has identified no abbreviated full form. However, the estimation of ache is depleted in the manuscript since no significant results were obtained regarding the parameter.

**Reviewer Comment 7**

In the material method section, under the subheading “Mass spectrometer instrument control by the analyst program” the last line “Precursor ions……………. confirmation” is not clear and hard to understand.

**Reply:** This section is a method that has previously developed by us for the estimation of OP metabolites on LCMS/MS in which precursor ions play a major role in the confirmation of mass of compounds to be identified. Further, the same method was applied here for identification of OP metabolites in the urine samples of children and the reference has been cited in the manuscript. (Page -9, Paragraph – 1)

**Reviewer Comment 8**

In the result section, in the second paragraph “ac-(methyl cyclopropyl) glycine” should be replaced with “α-methyl cyclopropyl glycines”.

**Reply:** As per the suggestions of the reviewer, α-methyl cyclopropyl glycines has been replaced for ac-(methyl cyclopropyl) glycine in the revised manuscript. (Page 10, Paragraph 3)

**Reviewer Comment 9**

In the result section, under the subheading “Isolation of MCPG”, the author should clearly define the respective Rf values for each sample.

**Reply:** The Rf values for each sample was incorporated and rewritten as per the reviewer suggestions in the revised manuscript. (Page 11, Paragraph 1)

**Reviewer Comment 10**

In the discussion section, in the first paragraph and second-line “Analysis……………. critically” should be rewritten.

**Reply:** As per the reviewer suggestions the lines were rewritten as, “The litchi fruit samples and biological (urine) samples of the children from Muzaffarpur were collected and analyzed in the present study to critically investigate the cause of the outbreak” in the revised manuscript. (Page 12, Paragraph 2)

**Reviewer Comment 11**

In the fourth paragraph of the discussion “toxics” should be replaced with “toxins”.

**Reply:** The word toxics has been replaced with toxins as per the review suggestions in the revised manuscript. (Page 12, Paragraph 3)

***Reviewer #3:***

**Reviewer Comment 1**:

MCPG is a well reported metabolite in litchi. I was just wondering if authors found any other toxic compounds in the litchi fruit.

**Reply:** We were focusing only on MCPG and pesticide metabolites in the present study and no other toxic compounds were identified. However, it would be interesting and innovative to look beyond and identify if there could be any other compounds that are toxic in litchi fruit.

**Reviewer Comment 2:**

Did authors find any OP pesticides in litchi fruit? If yes what are they?

**Reply:** We have extracted the litchi fruits to identify any pesticides but we could not find any significant levels of OP pesticides in any of the fruit samples.

**Reviewer Comment 3**:

I would suggest quantifying the MCPG in the litchi samples and quantify DEP in urine samples.

**Reply:** We thank the reviewer for their valuable comments and suggestions and the DEO metabolites in urine samples were quantified and the levels were found to be 3327 ɳg/mL which was identified in one urine sample. We could not quantify the level of MCPG as we could not get access to the standard of MCPG. (Page 12, Paragraph 1)
