# Supplementary material for: A recurring disease outbreak following litchi fruit consumption among children in Muzaffarpur, Bihar—A comprehensive investigation on factors of toxicity
Source: PLoS One. 2020 Dec 31;15(12):e0244798. doi: 10.1371/journal.pone.0244798 (PMC7774918; doi:10.1371/journal.pone.0244798)
Supplement: Attachment — Submitted filename: PONE-D-20-25356 R2- Responces.docx [file pone.0244798.s003.docx]

**Response to reviewer comments**

**Comment 1:** Improve the english of the manuscript

**Response:** We thank the editor and reviewers for their comments and help us in improving our manuscript. The English and grammar have been corrected using Editage services and the certificate has been attached with cover letter for your kind reference.

**Comment 2:** Explain how the present study findings are informative than previously published results

**Response:** Although similar studies were conducted previously, they focused on identifying MCPG, a known toxin found in litchi. However, the present study considered other sources of toxicity that can occur due to litchi consumption. The use of OP pesticides in Bihar for litchi cultivation called for a thorough investigation. Therefore, litchi fruit samples and urine samples from the children from Muzaffarpur were collected and analyzed in the present study to critically investigate and identify the toxic compounds (MCPG and OP pesticide metabolites), which may have caused the fatalities. The above has also been included in the revised manuscript.

**Comment 3:** Improve the quality of images

**Response:** The original images have been uploaded along with the revised manuscript.
